# SPIKE-Synchronization: a parameter-free and time-resolved coincidence detector with an intuitive multivariate extension

**DOI:** 10.1186/1471-2202-16-S1-P170

**Published:** 2015-12-18

**Authors:** Thomas Kreuz, Nebojsa Bozanic, Mario Mulansky

**Affiliations:** 1 Institute for Complex Systems, CNR, Sesto Fiorentino, Italy

## 

Techniques for recording large-scale neuronal spiking activity are developing very fast. This leads to an increasing demand for algorithms capable of analyzing large amounts of experimental spike train data. One of the most crucial and demanding tasks is the identification of similarity patterns with high temporal resolution and across different spatial scales. To address this task, in recent years three time-resolved measures of spike train synchrony have been proposed, event synchronization [1], the ISI-distance [2], and the SPIKE-distance [3].

Here we present SPIKE-synchronization [4], an improved and simplified extension of event synchronization with a more intuitive interpretation which holds for both the bivariate and the multivariate case. SPIKE-synchronization quantifies the degree of synchrony from the relative number of quasi-simultaneous appearances of spikes. Since it builds on the same bivariate and adaptive coincidence detection that was used for event synchronization, SPIKE-synchronization is parameter- and scale-free as well. This makes it easy to handle and allows for an objective estimation of neuronal synchronization. In contrast to the ISI- and the SPIKE-distance, SPIKE-synchronization is a measure of similarity. It is zero if and only if the spike trains do not contain any coincidences, and reaches one if and only if each spike in every spike train has one matching spike in all the other spike trains.

We investigate the properties of SPIKE-synchronization and compare it against other time-resolved measures such as the Peri-Stimulus Time Histogram (PSTH) and the ISI- and the SPIKE-distance [4,5]. We use simulated data to verify its usefulness and explore its performance on real data.

Together with the ISI-distance and the SPIKE-distance, SPIKE-Synchronization is implemented in both the Matlab-based graphical user interface SPIKY and the Python library PySpike [6]. Both packages provide ample documentation as well as platforms for user feedback. SPIKY even comes with an interactive Facebook page (https://www.facebook.com/SPIKYgui) and a YouTube channel (https://www.youtube.com/user/SPIKYgui1) which includes movies demonstrating both the measures and the GUI.
